# Exploring the inhibition mechanism of interleukin-1-beta in gouty arthritis by polygonum cuspidatum using network pharmacology and molecular docking: A review

**DOI:** 10.1097/MD.0000000000034396

**Published:** 2023-07-21

**Authors:** Xiao Ge, Yan Zhang, Rulu Fang, Jiaojiao Zhao, Jiyong Huang

**Affiliations:** a The Second Clinical Medical College of Zhejiang Chinese Medical University, Hangzhou, China; b Intensive Care Union, The Third Affiliated Hospital of Zhejiang Chinese Medical University, Hangzhou, China; c Department of Immunology and Rheumatology, The Second Affiliated Hospital of Zhejiang Chinese Medical University, Hangzhou, China.

**Keywords:** gouty arthritis, network pharmacology, polygonum cuspidatum

## Abstract

Polygonum cuspidatum (Huzhang, HZ) is one of the commonly used traditional Chinese medicines for treating gouty arthritis (GA), but the specific mechanism is not clear. This study employed network pharmacology and molecular docking techniques to examine the molecular mechanisms underlying the therapeutic effects of HZ on GA. The network pharmacology approach, including active ingredient and target screening, drug-compound-target-disease network construction, protein-protein interaction (PPI) networks, enrichment analysis, and molecular docking, was used to explore the mechanism of HZ against GA. Ten active ingredients of HZ were predicted to interact with 191 targets, 14 of which interact with GA targets. Network pharmacology showed that quercetin, physovenine, luteolin, and beta-sitosterol are the core components of HZ, and IL (interleukin)-1β, IL-6, and tumor necrosis factor (TNF) are the core therapeutic targets. The mechanism of HZ in GA treatment was shown to be related to the IL-17 signaling pathway, NOD-like receptor signaling pathway, and Toll-like receptor signaling pathway, and is involved in the inflammatory response, positive regulation of gene expression, cellular response to lipopolysaccharide, and other biological processes. Molecular docking showed that all four core compounds had good binding properties to IL-1β, with luteolin and beta-sitosterol showing better docking results than anakinra, suggesting that they could be used as natural IL-1β inhibitors in further experimental studies. The mechanism of action of HZ against GA has multi-target and multi-pathway characteristics, which provides an important theoretical basis for the study of the active ingredients of HZ as natural IL-1β inhibitors.

## 1. Introduction

Gouty arthritis (GA) is a disease characterized by disruption of purine metabolism and deposition of monosodium urate (MSU) crystals around various joints, which can lead to an inflammatory response in the affected joints.^[1]^ According to relevant reports, the global prevalence of GA is 0.1% to 10%.^[2]^ Along with the development of the condition, GA may also be combined with other medical diseases, such as nephropathy,^[3,4]^ complicating treatment and seriously affecting the patients’ quality of survival and prognosis. If not treated aggressively, there is risk of teratogenicity and disability.^[5]^ Clinically commonly used drugs for GA, such as colchicine, nonsteroidal anti-inflammatory drugs, glucocorticoids, and febuxostat, are effective but can have adverse effects such as liver function impairment, renal function impairment, and gastrointestinal reactions.^[6,7]^ It is also prone to relapse after drug discontinuation,^[8]^ and patient compliance is poor.

Traditional Chinese medicine (TCM) has characteristics such as excellent efficacy, affordability price, and few side effects in the prevention and treatment of GA. Unlike Western medicine, traditional Chinese medicine has the advantages of “multi-component, multi-target, and multi-pathway.” Therefore, the use of TCM to prevent and treat GA is a key research topic. Polygonum cuspidatum (Huzhang, HZ) is a perennial shrubby herb that is the rhizome of Polygonum cuspidatum, a plant belonging to the Polygonaceae family. It has the effects of clearing heat and detoxifying, promoting dampness and eliminating jaundice, dispersing blood stasis and relieving pain, resolving phlegm and relieving cough, and is often used to treat arthritis, hepatitis, jaundice, hyperlipidemia, and cough.^[9]^ Under the guidance of TCM theory, HZ is also used to treat GA with significant efficacy and fewer side effects. However, the mechanism of action of HZ medicinal substances against GA is unclear.

In vitro and in vivo studies have confirmed that interleukin-1-beta (IL-1β) plays an essential role in the formation and destruction of gout,^[10]^ and when the upstream pathway of IL-1β production is blocked, the level of IL-1β can be effectively lowered, alleviating gout attacks and reducing pain in patients.^[11]^ IL-1β is a critical cytokines in the acute inflammatory response to gout and can cause an inflammatory response. More importantly, it can simultaneously induce the expression of various pro-inflammatory cytokines such as interleukin-6, interleukin-8, and tumor necrosis factor-alpha, as well as adhesion molecules and chemokines, exacerbating the inflammatory response. Therefore, blocking or antagonizing IL-1β could be a new target for gout treatment. Clinically relevant therapeutic agents targeting gout-producing IL-1β have also been undertaken. Anakinra is a recombinant human IL-1 receptor antagonist approved for marketing by the US Food and Drug Administration. Although anakinra is not an Food and Drug Administration-approved indication for gout treatment, the 2012 American College of Rheumatology guidelines for treating gout still recommend anakinra for treating refractory gout.^[12–14]^ In summary, due to the enormous potential of HZ, we hope to bring more and safer clinical effects to treat patients with GA by exploring its mechanisms of action.

Network pharmacology is a method that integrates pharmacology, molecular biology, medicine, bioinformatics, and statistics and is suitable for studies related to herbal medicines with complex compositions.^[15]^ In this study, we first used the Traditional Chinese Medicine Systems Pharmacology (TCMSP) database to identify HZ’s effective ingredients and targets of HZ. Second, the GA genes were obtained using the Online Mendelian Inheritance in Man (OMIM) and Disgenet databases. Potential treatment targets were identified by the intersection of the 2 groups. Functionally enriched analysis at bioinformatics.com.cn. Cytospace software and the STRING database were used to produce the drug-component-target-disease network and protein-protein interaction (PPI) network. Finally, we used molecular coupling to verify the ability of the HZ active ingredient to bind to the target and compared it with anakinra. We used network pharmacology to reveal the therapeutic value and potential mechanisms of HZ in GA, hoping to provide more advice and guidance for the treatment of GA.

## 2. Materials and methods

### 2.1. Searching and screening of active compounds and related targets in HZ

The TCMSP (https://tcmsp-e.com/tcmsp.php)^[16]^ database was used to screen HZ compounds. Furthermore, oral bioavailability ≥ 30% and drug-likeness ≥ 0.18^[17]^ were used as screening conditions to identify the active compounds and related targets of HZ. The UniProt (https://www.uniprot.org/)^[18]^ database was used to translate the acquired targets into gene names. Complexes corresponding to nonhuman genes and nonhuman genes were deleted.

### 2.2. Acquisition of GA targets

“Gout” was used as a keyword to search the DisGeNET (https://www.disgenet.org/)^[19]^ database and the OMIM (https://omim.org/)^[20]^ database, and the same genes were deleted from both databases to obtain the final gout-related genes.

### 2.3. Construction of drug-component-target-disease network

To intuitively express the relationship between drugs and diseases, we introduced the obtained targets of HZ and GA into Cytoscape software (version 3.8.0)^[21]^ to obtain a drug-component-target-disease network. The lines indicate the presence of a relationship between them, and the nodes indicate relevant targets.

### 2.4. Acquisition of intersection targets

Drug targets were intersected with disease targets using Venny 2.1.0 (https://bioinfogp.cnb.csic.es/tools/venny/index.html), and the intersected targets were potential targets for HZ intervention in GA.

### 2.5. Construction of PPI network

The intersecting targets obtained were imported into the STRING 11.5 database (https://string-db.org/).^[22]^ Set the species as “Homo sapiens” and set a confidence level greater than 0.4. The free targets in the network were then hidden, and the protein-protein interaction (PPI) network was constructed. The obtained network diagram was then used to build a PPI network using Network Analysis in the Cytoscape software. Where lines represent relationships and nodes represent related targets. In this network, the lines indicate the existence of relationships between them, and the nodes indicate relevant targets.

### 2.6. Biological function and pathway enrichment analysis of core targets

The DAVID Bioinformatics Resources (https://david.ncifcrf.gov/)^[23]^ was used for enrichment analysis of the intersecting genes, and *P* values processed the data and visualized the results obtained using bioinformatics.com.cn.

### 2.7. Molecular docking

The 2D structure of the active components of HZ was downloaded from the PubChem (https://pubchem.ncbi.nlm.nih.gov/)^[24]^ database, and then the 2D structure was converted into a 3D structure using Chem3D software. In addition, the IL-1β (PDB DOI: 10.2210/pdb5R8Q/pdb) protein molecular structures were downloaded from the PDB database (https://www.rcsb.org/),^[25]^ and the receptor protein was routinely pretreated with PyMol (version 2.2.0) software for dehydration, hydrogenation, and removal of irrelevant ligands. Finally, molecular docking of the ligands to the target receptor was performed using the AutoDockTools software. To evaluate the docking results, we downloaded the IL-1 receptor antagonist anakinra (CID: 90470007) from the PubChem database as a ligand for molecular docking. Finally, the lowest binding energy results were selected separately and visualized using PyMol software.

## 3. Results

### 3.1. Active ingredients and targets of HZ

Using the TCMSP database, 10 active ingredients of HZ were obtained using oral bioavailability ≥ 30% and drug-likeness ≥ 0.18 as screening conditions. Additionally, we converted the targets corresponding to the active ingredients into their corresponding genes using the UniProt database. Besides, twenty-three non-human genes were deleted, and duplicate genes were removed to obtain 191 genes. Among them, quercetin had the highest number of targets (144 genes), followed by luteolin (54 genes), beta-sitosterol (34 genes), and physovenine (34 genes). The results of compound screening are shown in Table 1.

**Table 1:** Characteristics of eligible active compounds in HZ.

Molecule ID	Molecule name	Molecule weight	OB (%)	DL
MOL000098	Quercetin	302.25	46.43	0.28
MOL000006	Luteolin	286.25	36.16	0.25
MOL013287	Physovenine	262.34	106.21	0.19
MOL000358	Beta-sitosterol	414.79	36.91	0.75
MOL013281	6,8-Dihydroxy-7-methoxyxanthone	258.24	35.83	0.21
MOL000492	(+)-Catechin	290.29	54.83	0.24
MOL002268	Rhein	284.23	47.07	0.28
MOL013288	Picralinal	366.45	58.01	0.75
MOL002259	Physciondiglucoside	608.60	41.65	0.63
MOL002280	Torachrysone-8-O-Beta-D-(6′-oxayl)-Glucoside	480.46	43.02	0.74

DL = drug-likeness, HZ = polygonum cuspidatum, OB = oral bioavailability.

### 3.2. Targets of HZ for GA treatment

**Figure 1.**
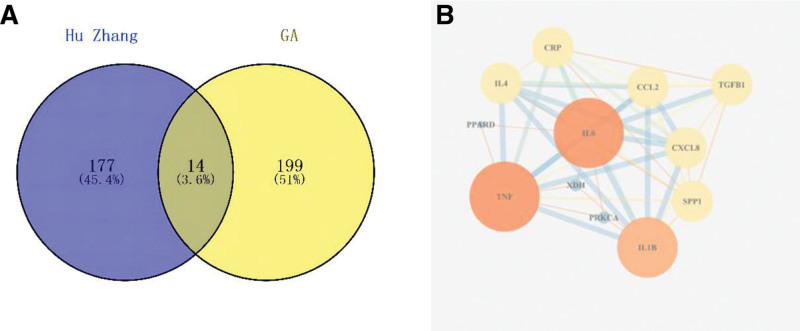
Potential therapeutic targets and PPI network map of HZ for GA. (a) The Venny results of potential therapeutic targets of HZ for GA. (b) The PPI network map of 12 core targets. GA = gouty arthritis, HZ = polygonum cuspidatum, PPI = protein-protein interaction.

The OMIM and DisGeNet databases were used to identify GA-related disease targets. The 2 databases were pooled and duplicate values were removed to obtain a total of 213 disease targets, which were intersected with drug targets to obtain 14 potential therapeutic targets. Venny 2.1.0 was used to import the GA disease targets and HZ targets to create a Venny diagram. The interaction between HZ and GA targets is shown in Figure 1A.

### 3.3. Construction of PPI network diagram and core targets screening

The OMIM and DisGeNet databases were used to identify GA-related disease targets. Moreover, the 2 databases were pooled, and duplicate values were removed to obtain 213 disease targets, which were intersected with drug targets to obtain 14 potential therapeutic targets. Finally, we used Venny 2.1.0 to import the GA disease and HZ targets to create a Venny diagram. The interaction between HZ and GA targets is shown in Figure 1B.

### 3.4. HZ-component-target-GA network

**Figure 2.**
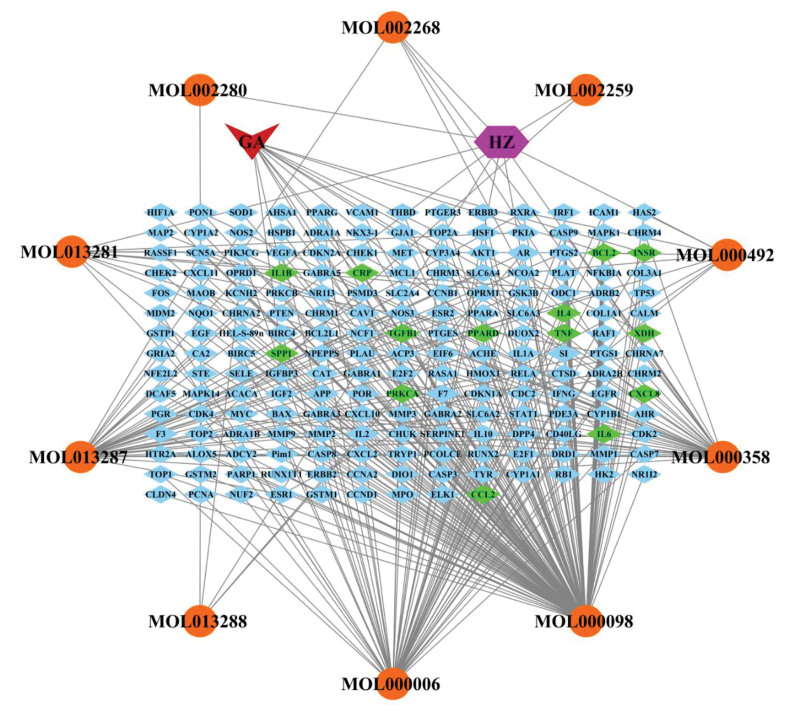
The HZ-compound-target-GA network. The purple node represents HZ. The red node represents GA. Orange nodes represent active compounds. Blue nodes represent targets. Green nodes represent potential core targets of HZ treatment for GA. Gray lines represent interconnections between nodes and nodes. GA = gouty arthritis, HZ = polygonum cuspidatum.

Potential targets and active compounds were imported into the Cytoscape software to produce an HZ-component-target-GA network map. A total of 203 nodes and 319 edges were obtained from the network diagram. All nodes were sorted by degree value using the Network Analyzer function in the software tool, with the most significant compound being quercetin (degree: 145), followed by luteolin (degree: 55), physovenine (degree: 35), and beta-sitosterol (degree: 35). Therefore, they are potentially vital compounds for the treatment of HZ with GA. The details of the HZ-compound-target-GA network are shown in Figure 2.

### 3.5. Biological function and pathway enrichment analyses of core targets.

**Figure 3.**
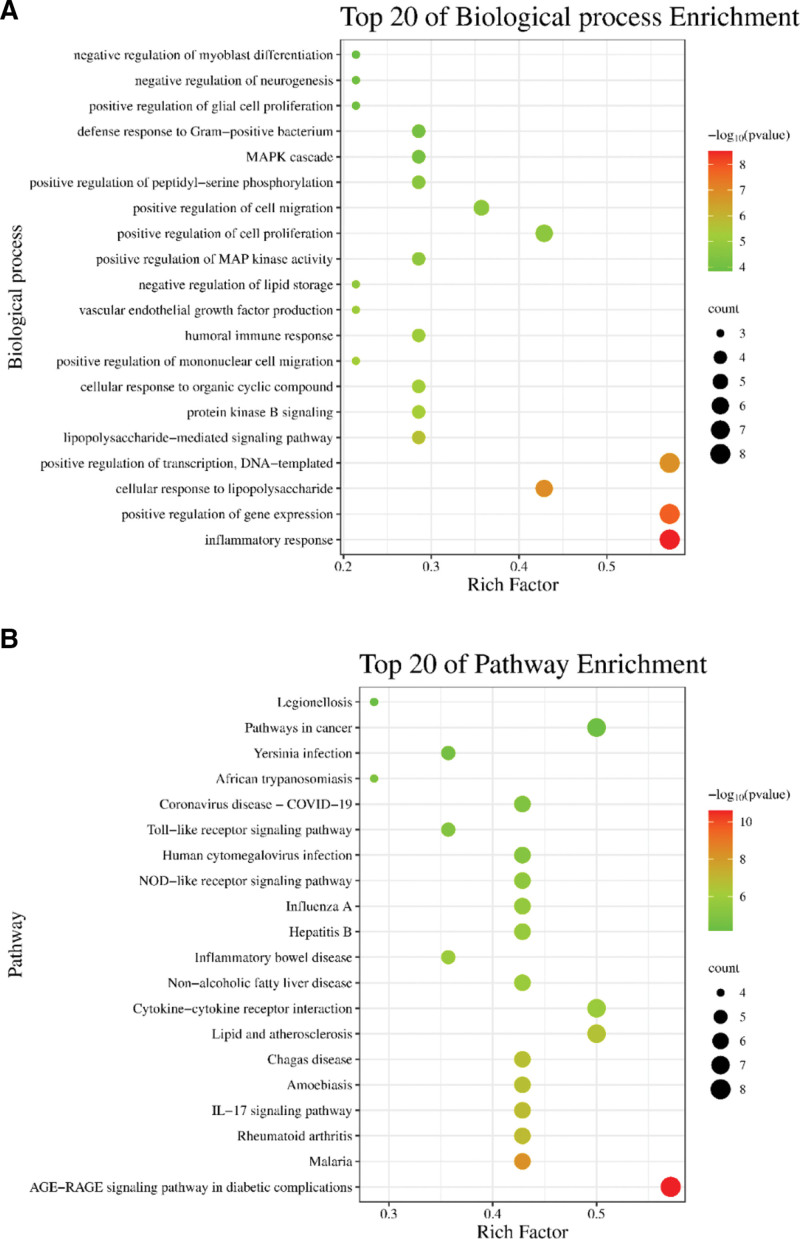
Biological process and pathway analyses from bioinformatics data. (A) Biological process enrichment analysis of therapeutic genes. (B) Pathway enrichment analysis of therapeutic genes.

We imported potential targets into DAVID Bioinformatics Resources for analysis to investigate the biological mechanism of GA treatment with HZ. We then filtered the results to the top 20 biological process and pathway enrichments based on *P* values. We then imported the results into bioinformatics.com.cn to produce an advanced bubble chart. The results show that the biological processes of the potential core targets were mainly involved in the inflammatory response, positive regulation of gene expression, cellular response to lipopolysaccharide, positive regulation of DNA-templated transcription, lipopolysaccharide-mediated signaling pathway, protein kinase B signaling, cellular response to organic cyclic compounds, positive regulation of mononuclear cell migration, humoral immune response, vascular endothelial growth factor production, etc. In addition, the pathway enrichment results mainly included the AGE-RAGE signaling pathway in diabetic complications, Malaria, Rheumatoid arthritis, interleukin-17 (IL-17) signaling pathway, Amoebiasis, Chagas disease, lipid and atherosclerosis, cytokine-cytokine receptor interaction, nonalcoholic fatty liver disease, Inflammatory bowel disease, etc. Biological process enrichment is shown in Figure 3A. Pathway enrichment is shown in Figure 3B.

### 3.6. Molecular docking

**Figure 4.**
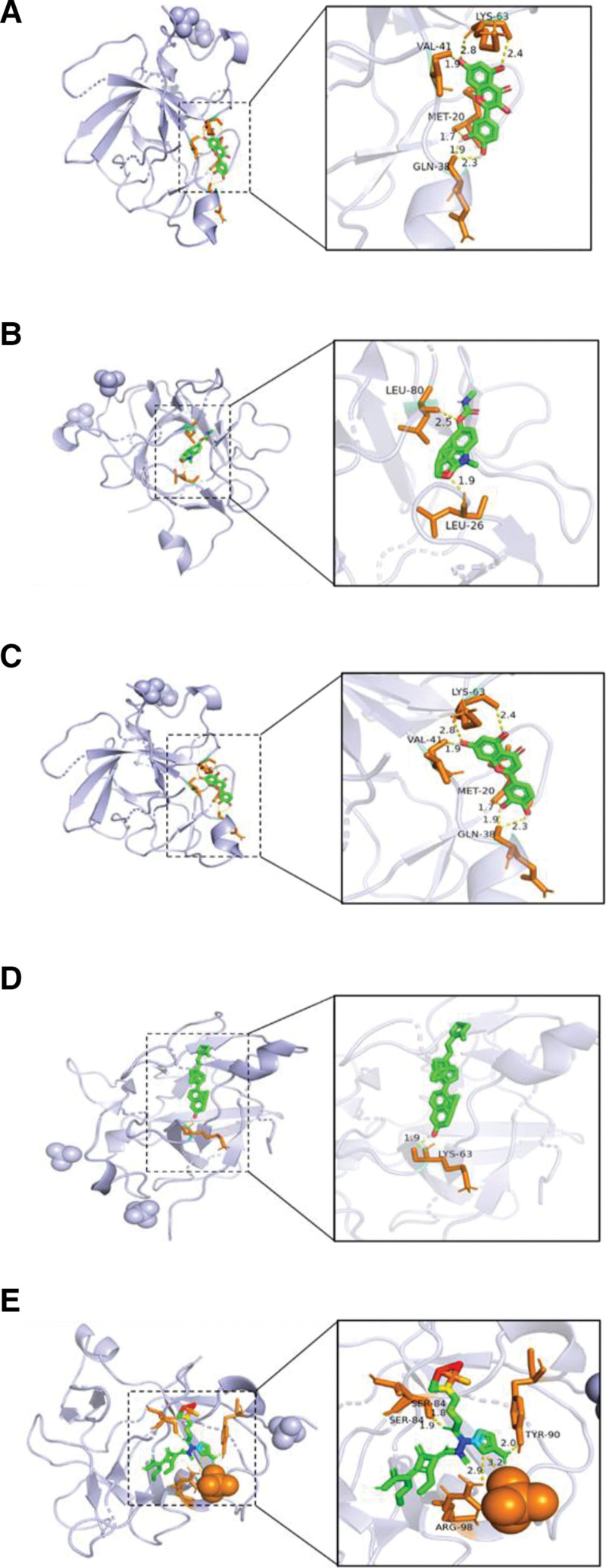
The diagram of the binding of quercetin (A), physovenine (B), luteolin (C), beta-sitosterol (D), and anakinra (E) with IL-1β.

Molecular docking of quercetin, luteolin, physovenine, and beta-sitosterol with IL-1β. The lower the docking binding energy of the compound to the target molecule, the more tightly the 2 molecules are bound. Binding energies < −4.25 kcal-mol-1 indicate some binding function between the active ingredient and the protein, good binding function at binding energies < −5.0 kcal-mol-1, and powerful binding function at binding energies < −7.0 kcal-mol-1.^[26]^ In addition, we downloaded the two-dimensional structure of the IL-1 receptor antagonist anakinra from Pub Chem for molecular docking to compare it with the 4 core components of HZ. The results showed that the binding energies of all 5 compounds were greater than 7.0 kcal-mol-1, indicating a strong affinity for IL-1β. Among them, the lowest binding energy of Luteolin and Beta-sitosterol was significantly lower than that of anakinra. The compound binding energies are presented in Table 2. The binding diagrams of quercetin, luteolin, physovenine, beta-sitosterol, and anakinra with IL-1β protein structure are shown in Figure 4.

**Table 2:** Results of molecular docking.

Target	Compound	Binding energy (kcal·mol^−1^)
IL-1β	Quercetin	−7.64
	Physovenine	−8.07
	Luteolin	−10.57
	Beta-sitosterol	−12.22
	Anakinra	−8.59

IL-1β = interleukin-1-beta.

## 4. Discussion

GA is a common heterogeneous disease characterized by purine metabolism disorders and elevated serum uric acid levels, which can lead to joint dysfunction.^[27]^ The pathogenesis of GA is complex, as MSU can be deposited in cartilage, synovium, and surrounding tissues when the concentration of uric acid is too high, exceeding the solubility of uric acid and becoming supersaturated. The deposited MSU irritates the synovial membrane of the joint, resulting in pathological reactions such as dilatation of synovial blood vessels, increased permeability, and leukocyte exudation, resulting in acute inflammatory symptoms of GA such as joint redness, swelling, and pain. IL-1β plays an essential role in GA as a critical cytokine in the inflammatory response. Related studies^[28,29]^ have pointed out that when MSU crystals stimulate a GA episode, they cause a massive release of IL-1β and upregulate the levels of neutrophils, which are associated inflammatory mediators. Excess neutrophils in the blood are selectively adsorbed to the surface of endothelial cells and enter the joint space through blood circulation, triggering an intense inflammatory response. It follows that IL-1β is an essential factor in GA attacks.^[30]^ In the present study, we revealed that HZ’s potential targets for GA treatment interact with each other through a network pharmacological approach. In other words, the richness of active ingredients in HZ makes it possible to achieve a multi-target synergistic effect in anti-GA. Furthermore, the PPI network map (Fig. 1B) revealed that HZ could treat GA via IL-1β.

The HZ-component-target-GA network diagram shows 10 active ingredients of HZ, of which 4 are core components in the fight against gout (Fig. 2). Quercetin, physovenine, luteolin, and beta-sitosterol are the active ingredients of various plants with various pharmacological effects, including antioxidant, anti-inflammatory, and immunomodulatory effects. Studies have shown that quercetin can act as a natural extract to reduce the inflammatory response by inhibiting xanthine oxidase, thereby reducing the levels of reactive oxygen species produced during the inflammatory process. Feng et al^[31]^ found that quercetin can reduce GA-induced ankle swelling and bone destruction in rats, suggesting that quercetin is a valuable alternative drug for treating GA. Previous studies have shown that luteolin can reduce serum uric acid levels in hyperuricemic mice by inhibiting the activity of hepatic xanthine oxidase and significantly reducing MSU-induced paw edema and IL-1β levels, making it a promising drug for the treatment of GA.^[32]^ In addition, beta-sitosterol has been shown to reduce MSU-induced paw edema in mice.^[33]^ There are no relevant studies on physovenine for the treatment of gout, which suggests that it could also be a candidate for subsequent experimental studies.

The biological process and pathway enrichment analysis of core targets revealed that HZ treatment of GA is multi-target and multi-pathway in nature and involves multiple biological processes (Fig. 3). Among them, the close pathways related to gout mainly contain the IL-17, NOD-like receptor, and Toll-like receptor signaling pathways. Biological processes include an inflammatory response, positive regulation of gene expression, cellular response to lipopolysaccharide, positive regulation of transcription, DNA-templated, and lipopolysaccharide-mediated signaling pathways. These biological functions and pathways are associated with the onset and development of GA, which may be the mechanism by which HZ treats GA. One of these pathways is the IL-17 signaling pathway, which is essential for the development of GA. IL-17 is mainly produced by T helper 17 cells (Th17) and acts as a pro-inflammatory factor that mediates inflammation.^[34]^ A study showed that serum levels of IL-17 in GA patients were significantly elevated during the early onset of gout symptoms and then gradually decreased as the symptoms decreased.^[35]^ Moreover, IL-17 cannot only exist as a pro-inflammatory factor but also stimulate various cells, such as epithelial and endothelial cells, to produce inflammatory factors that produce cytokines such as interleukin-6 and interleukin-8, leading to an inflammatory response. Recent studies have shown that IL-1β plays a vital role in the differentiation of IL-17-γδ T cells into IL-17+γδ T cells, suggesting that IL-17 may be a downstream pro-inflammatory cytokine of IL-1β.^[36,37]^ The NOD-like receptor signaling pathway and Toll-like receptor signaling pathway contain essential targets such as Toll-like receptor 4 (TRL4), Toll-like receptor 2 (TRL2), nuclear factor-kappa B dependent, and NOD-like receptor protein 3 inflammasome, and regulating these 2 pathways or targets can improve the inflammatory response in GA.^[29,38–40]^ This evidence suggests that these model pathways play an important role in the development of GA and that regulation of the relevant courses or targets can effectively control disease progression. Therefore, HZ treats GA through multiple biological functions and pathways.

Finally, we validated the core components of HZ with IL-1β using a docking technique. The results showed that quercetin, physovenine, luteolin, and beta-sitosterol showed good binding activity to the target protein (Fig. 4). We also compared them with anakinra and found that luteolin and beta-sitosterol bound better than anakinra (see results in Table 2). This suggests that these 2 compounds are promising natural IL-1β inhibitors. However, they need to be investigated further experimentally.

## 5. Conclusion

Network pharmacology plays an important role in the elucidation of the mechanisms of drug action. However, the present study had some limitations. On the one hand, this study screened the core targets under certain conditions, so potential genes outside the states may have been missed. Furthermore, these genes may also have some value. On the other hand, the results obtained in this study need to be further confirmed by experiments.

Overall, we validated the core components of HZ with IL-1β using a molecular docking technique, and the results showed that quercetin, physovenine, luteolin, and beta-sitosterol showed good binding activity to the target protein. We also compared these with anakinra and found that luteolin and beta-sitosterol were better. This suggests that these 2 compounds are promising natural IL-1β inhibitors, but they need to be investigated further experimentally.

## Author contributions

**Conceptualization:** Xiao Ge, Jiaojiao Zhao, Jiyong Huang.

**Data curation:** Xiao Ge, Jiaojiao Zhao.

**Formal analysis:** Xiao Ge, Yan Zhang.

**Investigation:** Xiao Ge, Rulu Fang.

**Methodology:** Xiao Ge, Yan Zhang.

**Writing – original draft:** Xiao Ge, Rulu Fang.

**Writing – review & editing:** Jiyong Huang.

## References

[1] Vedder D Walrabenstein W Heslinga M. Dietary interventions for gout and effect on cardiovascular risk factors: a systematic review. Nutrients. 2019;11:2955. 31817107 10.3390/nu11122955 PMC6950134

[2] Kuo CF Grainge MJ Zhang W. Global epidemiology of gout: prevalence, incidence and risk factors. Nat Rev Rheumatol. 2015;11:649–62. 26150127 10.1038/nrrheum.2015.91

[3] Singh JA. Gout and comorbidity: a nominal group study of people with gout. Arthritis Res Ther. 2017;19:204. 28915838 10.1186/s13075-017-1416-8 PMC5603046

[4] Drivelegka P Sigurdardottir V Svärd A. Comorbidity in gout at the time of first diagnosis: sex differences that may have implications for dosing of urate lowering therapy. Arthritis Res Ther. 2018;20:108. 29855389 10.1186/s13075-018-1596-x PMC5984404

[5] Pascual E Andrés M Vázquez-Mellado J. Severe gout: strategies and innovations for effective management. Joint Bone Spine. 2017;84:541–6. 27932279 10.1016/j.jbspin.2016.10.004

[6] Rey A Batteux B Laville SM. Acute kidney injury associated with febuxostat and allopurinol: a post-marketing study. Arthritis Res Ther. 2019;21:229. 31703711 10.1186/s13075-019-2011-y PMC6842268

[7] Du X Zhao L Yang Y. Investigation of the mechanism of action of Porana sinensis Hemsl. against gout arthritis using network pharmacology and experimental validation. J Ethnopharmacol. 2020;252:112606. 31988013 10.1016/j.jep.2020.112606

[8] Day R Nguyen A Graham G. Better outcomes for patients with gout. Inflammopharmacology. 2020;28:1395–400. 32095979 10.1007/s10787-020-00694-7

[9] Chinese Pharmacopoeia Committee, Chinese Pharmacopoeia Commission: Part I. Beijing, China: China Medical Science and Technology Press; 2010;194–5.

[10] Mian W Zhang M Ma Y. Chaetocin attenuates gout in mice through inhibiting HIF-1α and NLRP3 inflammasome-dependent IL-1β secretion in macrophages. Arch Biochem Biophys. 2019;670:94–103. 31255694 10.1016/j.abb.2019.06.010

[11] Goldberg EL Asher JL Molony RD. β-Hydroxybutyrate Deactivates Neutrophil NLRP3 Inflammasome to Relieve Gout Flares. Cell Rep. 2017;18:2077–87. 28249154 10.1016/j.celrep.2017.02.004 PMC5527297

[12] Thueringer JT Doll NK Gertner E. Anakinra for the treatment of acute severe gout in critically ill patients. Semin Arthritis Rheum. 2015;45:81–5. 25795473 10.1016/j.semarthrit.2015.02.006

[13] Calvo-Aranda E Sanchez-Aranda FM. Efficacy of subcutaneous tocilizumab in a patient with severe gout refractory to anakinra. Rheumatology (Oxford). 2021;60:e375–7. 34009241 10.1093/rheumatology/keab383

[14] Jeria-Navarro S Gomez-Gomez A Park HS. Effectiveness and safety of anakinra in gouty arthritis: a case series and review of the literature. Front Med (Lausanne). 2023;9:1089993. 36714095 10.3389/fmed.2022.1089993 PMC9877612

[15] Ru J Li P Wang J. TCMSP: a database of systems pharmacology for drug discovery from herbal medicines. J Cheminform. 2014;6:13. 24735618 10.1186/1758-2946-6-13 PMC4001360

[16] Zhao L Zhang H Li N. Network pharmacology, a promising approach to reveal the pharmacology mechanism of Chinese medicine formula. J Ethnopharmacol. 2023;309:116306. 36858276 10.1016/j.jep.2023.116306

[17] Li J Zhao P Li Y. Systems pharmacology-based dissection of mechanisms of Chinese medicinal formula Bufei Yishen as an effective treatment for chronic obstructive pulmonary disease. Sci Rep. 2015;5:15290. 26469778 10.1038/srep15290 PMC4606809

[18] Soudy M Anwar AM Ahmed EA. UniprotR: Retrieving and visualizing protein sequence and functional information from Universal Protein Resource (UniProt knowledgebase). J Proteomics. 2020;213:103613. 31843688 10.1016/j.jprot.2019.103613

[19] Piñero J Queralt-Rosinach N Bravo A. DisGeNET: a discovery platform for the dynamical exploration of human diseases and their genes. Database (Oxford). 2015;2015:bav028. 25877637 10.1093/database/bav028 PMC4397996

[20] Amberger JS Bocchini CA Scott AF. OMIM.org: leveraging knowledge across phenotype-gene relationships. Nucleic Acids Res. 2019;47:D1038–43. 30445645 10.1093/nar/gky1151 PMC6323937

[21] Otasek D Morris JH Bouças J. Cytoscape Automation: empowering workflow-based network analysis. Genome Biol. 2019;20:185. 31477170 10.1186/s13059-019-1758-4 PMC6717989

[22] Szklarczyk D Gable AL Nastou KC. The STRING database in 2021: customizable protein-protein networks, and functional characterization of user-uploaded gene/measurement sets. Nucleic Acids Res. 2021;49:D605–12. 33237311 10.1093/nar/gkaa1074 PMC7779004

[23] Huang da W Sherman BT Lempicki RA. Systematic and integrative analysis of large gene lists using DAVID bioinformatics resources. Nat Protoc. 2009;4:44–57. 19131956 10.1038/nprot.2008.211

[24] Kim S Chen J Cheng T. PubChem 2023 update. Nucleic Acids Res. 2023;51:D1373–80. 36305812 10.1093/nar/gkac956 PMC9825602

[25] Bittrich S Rose Y Segura J. RCSB Protein Data Bank: improved annotation, search and visualization of membrane protein structures archived in the PDB. Bioinformatics. 2022;38:1452–4. 34864908 10.1093/bioinformatics/btab813 PMC8826025

[26] Hsin KY Ghosh S Kitano H. Combining machine learning systems and multiple docking simulation packages to improve docking prediction reliability for network pharmacology. PLoS One. 2013;8:e83922. 24391846 10.1371/journal.pone.0083922 PMC3877102

[27] Choi HK McCormick N Yokose C. Excess comorbidities in gout: the causal paradigm and pleiotropic approaches to care. Nat Rev Rheumatol. 2022;18:97–111. 34921301 10.1038/s41584-021-00725-9

[28] Wang X Chen D. Purinergic regulation of neutrophil function. Front Immunol. 2018;9:399. 29545806 10.3389/fimmu.2018.00399 PMC5837999

[29] Xu L Liu X Zhang Y. Tanshinone IIA improves acute gouty arthritis in rats through regulating neutrophil activation and the NLRP3 inflammasome. Dis Markers. 2022;2022:5851412. 36578443 10.1155/2022/5851412 PMC9792249

[30] So A Dumusc A Nasi S. The role of IL-1 in gout: from bench to bedside. Rheumatology (Oxford). 2018;57(suppl_1):i12–9. 29272514 10.1093/rheumatology/kex449

[31] Feng W Zhong XQ Zheng XX. Study on the effect and mechanism of quercetin in treating gout arthritis. Int Immunopharmacol. 2022;111:109112. 35932610 10.1016/j.intimp.2022.109112

[32] Lin Y Liu PG Liang WQ. Luteolin-4’-O-glucoside and its aglycone, two major flavones of Gnaphalium affine D. Don, resist hyperuricemia and acute gouty arthritis activity in animal models. Phytomedicine. 2018;41:54–61. 29519319 10.1016/j.phymed.2018.02.002

[33] de Souza MR de Paula CA Pereira de Resende ML. Pharmacological basis for use of Lychnophora trichocarpha in gouty arthritis: anti-hyperuricemic and anti-inflammatory effects of its extract, fraction and constituents. J Ethnopharmacol. 2012;142:845–50. 22732730 10.1016/j.jep.2012.06.012

[34] Beringer A Noack M Miossec P. IL-17 in chronic inflammation: from discovery to targeting. Trends Mol Med. 2016;22:230–41. 26837266 10.1016/j.molmed.2016.01.001

[35] Liu Y Zhao Q Yin Y. Serum levels of IL-17 are elevated in patients with acute gouty arthritis. Biochem Biophys Res Commun. 2018;497:897–902. 29476737 10.1016/j.bbrc.2018.02.166

[36] Kuwabara T Ishikawa F Kondo M. The role of IL-17 and related cytokines in inflammatory autoimmune diseases. Mediators Inflamm. 2017;2017:3908061. 28316374 10.1155/2017/3908061 PMC5337858

[37] Tabarkiewicz J Pogoda K Karczmarczyk A. The role of IL-17 and Th17 lymphocytes in autoimmune diseases. Arch Immunol Ther Exp (Warsz). 2015;63:435–49. 26062902 10.1007/s00005-015-0344-z PMC4633446

[38] Xu H Zhang B Chen Y. Type II collagen facilitates gouty arthritis by regulating MSU crystallisation and inflammatory cell recruitment. Ann Rheum Dis. 2023;82:416–27. 36109143 10.1136/ard-2022-222764

[39] Xu H Chen J Chen P. Costunolide covalently targets NACHT domain of NLRP3 to inhibit inflammasome activation and alleviate NLRP3-driven inflammatory diseases. Acta Pharm Sin B. 2023;13:678–93. 36873170 10.1016/j.apsb.2022.09.014 PMC9978959

[40] Sun X Li P Qu X. Isovitexin alleviates acute gouty arthritis in rats by inhibiting inflammation via the TLR4/MyD88/NF-κB pathway. Pharm Biol. 2021;59:1326–33. 34582722 10.1080/13880209.2021.1979595 PMC8480722

